# A Phenotype-Driven Approach to Generate Mouse Models with Pathogenic mtDNA Mutations Causing Mitochondrial Disease

**DOI:** 10.1016/j.celrep.2016.08.037

**Published:** 2016-09-13

**Authors:** Johanna H.K. Kauppila, Holly L. Baines, Ana Bratic, Marie-Lune Simard, Christoph Freyer, Arnaud Mourier, Craig Stamp, Roberta Filograna, Nils-Göran Larsson, Laura C. Greaves, James B. Stewart

**Affiliations:** 1 Department of Mitochondrial Biology, Max Planck Institute for Biology of Ageing, Cologne 50931, Germany; 2 Newcastle University LLHW Centre for Ageing and Vitality, Newcastle University, Newcastle upon Tyne NE2 4HH, UK; 3 Department of Medical Biochemistry and Biophysics, Karolinska Institutet, Stockholm 17177, Sweden; 4 Wellcome Trust Centre for Mitochondrial Research, Institute of Neuroscience, Newcastle University, Newcastle upon Tyne NE2 4HH, UK

## Abstract

Mutations of mtDNA are an important cause of human disease, but few animal models exist. Because mammalian mitochondria cannot be transfected, the development of mice with pathogenic mtDNA mutations has been challenging, and the main strategy has therefore been to introduce mutations found in cell lines into mouse embryos. Here, we describe a phenotype-driven strategy that is based on detecting clonal expansion of pathogenic mtDNA mutations in colonic crypts of founder mice derived from heterozygous mtDNA mutator mice. As proof of concept, we report the generation of a mouse line transmitting a heteroplasmic pathogenic mutation in the alanine tRNA gene of mtDNA displaying typical characteristics of classic mitochondrial disease. In summary, we describe a straightforward and technically simple strategy based on mouse breeding and histology to generate animal models of mtDNA-mutation disease, which will be of great importance for studies of disease pathophysiology and preclinical treatment trials.

## Highlights

• We present a method to isolate and identify pathogenic mtDNA mutations in mice
• We describe a mouse with a pathogenic mutation in the mitochondrial *tRNA*^*ALA*^ gene
• The mice display disrupted mitochondrial translation as a result of the mutation
• The mice display molecular and histochemical symptoms of human mitochondrial disease

## Graphical Abstract

**Figure:**
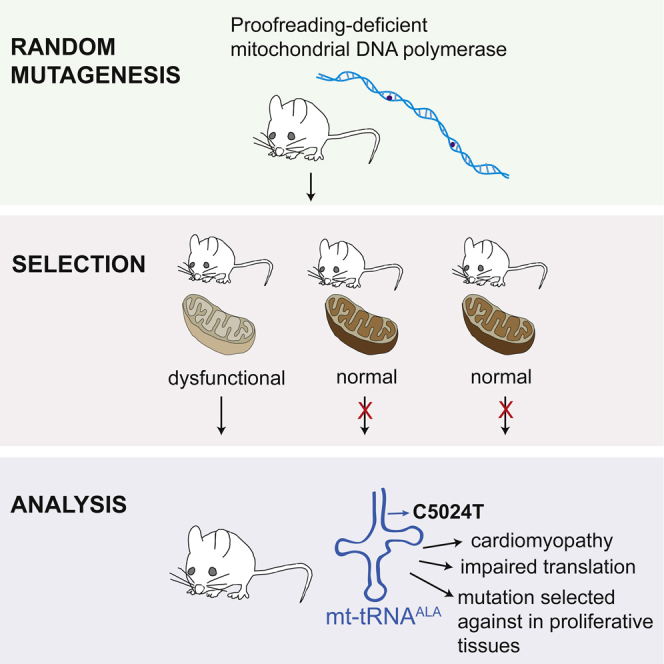

## Teaser

Kauppila et al. describe a phenotype-based screen in live mice to generate mouse models with pathogenic mtDNA mutations. As proof of concept, they present a mouse with a mutation in the mitochondrial *tRNA*^*ALA*^ gene that displays molecular and histochemical symptoms of human mitochondrial disease.

## Introduction

The biogenesis of the oxidative phosphorylation system is critically dependent on mtDNA expression (Larsson et al., 1998). Consequently, mutations of mtDNA are an important cause of mitochondrial disease (Greaves et al., 2012, Keogh and Chinnery, 2015) and are suggested to participate in the aging process (Larsson, 2010). The pathophysiology of mtDNA disease is poorly understood, as emphasized by the observation that different types of mutations affecting tRNA genes often cause distinct clinical phenotypes (Tyynismaa and Suomalainen, 2009). Single large deletions of mtDNA typically remove one or several tRNA genes and lead to a multisystem disease in children (Larsson et al., 1990, Rötig et al., 1990) or chronic progressive external ophthalmoplegia (CPEO) with additional neuromuscular symptoms in adults (Moraes et al., 1989). The A8344G mutation in the *tRNA*^*LYS*^ (*mt-Tk*) gene causes myoclonus epilepsy and ragged-red fibers syndrome (MERRF) (Shoffner et al., 1990), whereas the A3243G mutation in the *tRNA*^*LEU(UUR)*^ (*mt-Tl1*) gene can cause a spectrum of clinical phenotypes, including maternally inherited diabetes and deafness (MIDD) (Nesbitt et al., 2013); mitochondrial myopathy, encephalopathy, lactic acidosis, and stroke-like episodes syndrome (MELAS) (Goto et al., 1990); or CPEO (Moraes et al., 1992). The genotype-phenotype correlations exemplified above are not at all understood and are particularly enigmatic if one considers that all mutations affecting tRNA genes should have the common effect of impairing mitochondrial translation (Larsson and Clayton, 1995). In vivo experimental approaches are clearly needed to better understand the pathophysiology of different types of mtDNA mutations. Unfortunately, robust procedures for transfection of mammalian mitochondria have so far not been developed, and attempts to generate mouse models have therefore been focused on introducing pre-existing mtDNA mutations from cell lines or somatic tissues into mice. One important strategy to generate such transmitochondrial mice has been to perform fusion between female karyotype embryonic stem cells (ESCs) and cytoplasmic fragments containing mitochondria with pathogenic mtDNA mutations. The first pathogenic mutation introduced into mice with this technique affected the *lrRNA* gene and conferred resistance to chloramphenicol toxicity (Levy et al., 1999, Marchington et al., 1999, Watanabe et al., 1978). The technique was subsequently amended by introducing a step to remove the endogenous mitochondria in the ESCs by treatment with the toxin rhodamine 6G prior to the introduction of exogenous mitochondria (Sligh et al., 2000). Although this procedure improved the efficiency for introduction of mutant mtDNA, animals carrying high levels of the *lrRNA* gene mtDNA mutation died as embryos or as newborn pups shortly after birth. Pathogenic mtDNA mutations have also been introduced into mice by fusion of enucleated cytoplasm to fertilized oocytes. This method has successfully been used to introduce duplicated/deleted mtDNA into mice (Inoue et al., 2000, Nakada et al., 2004), whereas the ESC method has been used to introduce mtDNA with mutations affecting protein-coding genes (Fan et al., 2008) or tRNA genes (Shimizu et al., 2014, Shimizu et al., 2015). Some of these transmitochondrial mice develop mitochondrial disease phenotypes such as cardiomyopathy, muscle atrophy, and anemia (Fan et al., 2008, Inoue et al., 2000, Nakada et al., 2004, Shimizu et al., 2015, Sligh et al., 2000). Unfortunately, a clear drawback of the cytoplasmic fusion strategy is that it is very laborious and that the choice of mutations is limited to those that already exist in cell lines or somatic tissues. Furthermore, some of the introduced mutations cannot be propagated in mice (Fan et al., 2008) because of strong purifying selection in the maternal germline (Stewart and Larsson, 2014).

The mtDNA mutator mice are homozygous for a knockin mutation in the gene encoding the catalytic subunit of the mtDNA polymerase (*PolgA*^MUT/MUT^) and express a proofreading-deficient enzyme with much reduced 3′–5′ exonucleolytic activity (Ross et al., 2013, Trifunovic et al., 2004). As a consequence, the *PolgA*^MUT/MUT^ mice have extensive somatic and germline mutagenesis of mtDNA and will transmit mutated mtDNA through the germline (Ross et al., 2013, Stewart et al., 2008). Mouse lines derived by breeding *PolgA*^MUT/MUT^ females have been valuable tools to study purifying selection and have clarified that mutations causing amino acid substitutions in respiratory chain subunits are strongly selected against in the maternal germline (Stewart and Larsson, 2014, Stewart et al., 2008). In contrast, tRNA gene mutations are better tolerated and mainly undergo purifying selection during embryonic development (Freyer et al., 2012). Unfortunately, the breeding of *PolgA*^MUT/MUT^ mice results in mouse lines that have many linked mutations in the same mtDNA molecule, which makes it difficult to establish firm genotype-phenotype correlations. These models are therefore of limited use when it comes to understanding the enigmatic pathophysiological effects of single specific mtDNA mutations.

To overcome these limitations, we describe here a technically simple and straightforward screening approach to create mice with pathogenic mtDNA mutations. In a first step, heterozygous mtDNA-mutator (*PolgA*^+/MUT^) females are bred to establish mouse lines with a wild-type nuclear background that each carries a very limited number of mtDNA mutations. In a second step, the founder individual of each line is sacrificed after having established a maternal mouse line and colonic crypts are analyzed by enzyme histochemistry to detect mosaic cytochrome *c* oxidase (COX) deficiency. In the third step, laser-capture dissection and mtDNA sequencing are performed on single colonic crypts to identify the pathogenic mtDNA mutation and establish its pathogenicity. Finally, identified mouse lines, where the founder mouse harbors a specific heteroplasmic pathogenic mtDNA mutation, are bred as maternal lines and extensively characterized. As a proof of principle, we describe here the creation of a mouse model for mitochondrial disease caused by a heteroplasmic C5024T mutation in the *tRNA*^ALA^ (*mt-Ta*) gene of mtDNA, which recapitulates important aspects of human mitochondrial disease.

## Results

### Breeding of Mouse Lines to Isolate Single Pathogenic mtDNA Mutations

**Figure 1:**
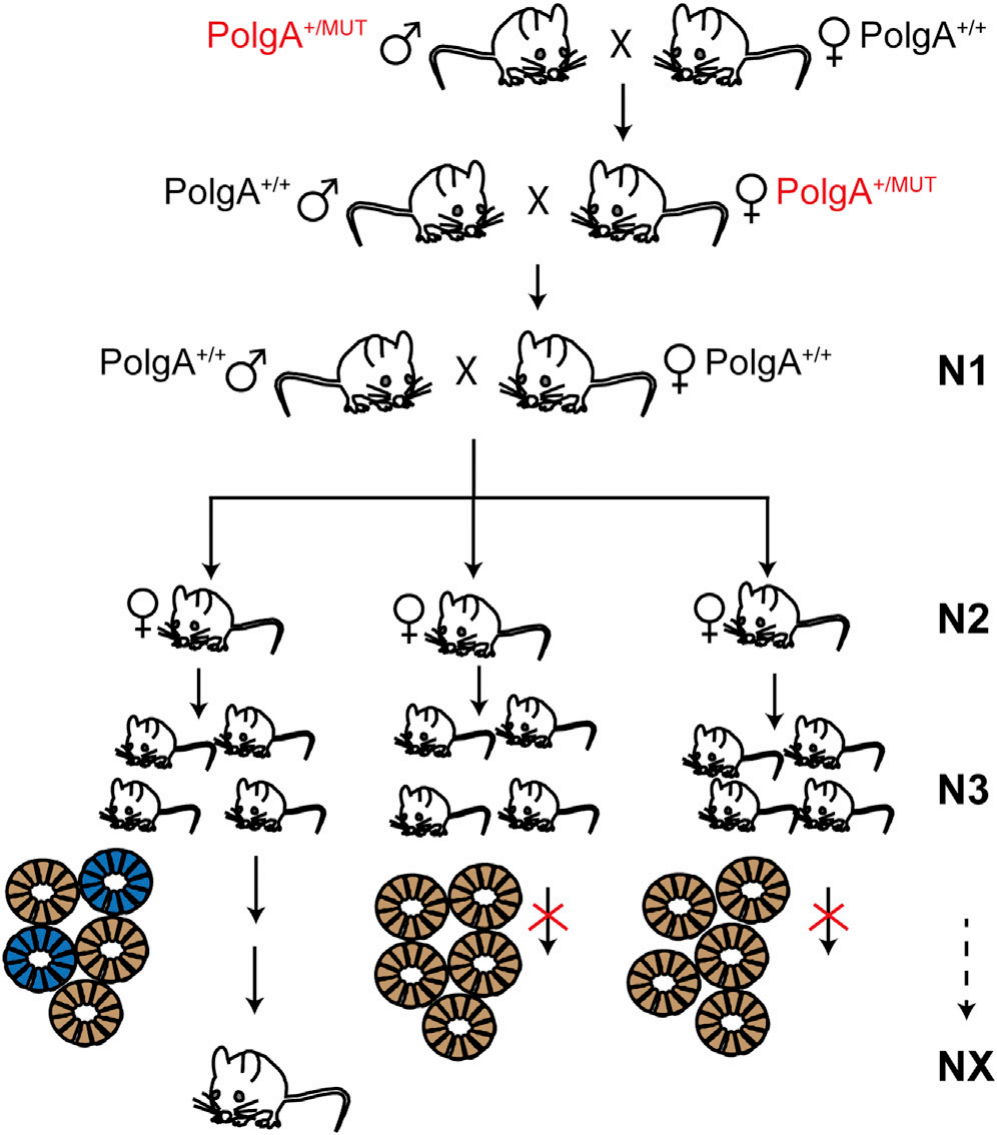
Breeding Scheme to Generate Mouse Lines Transmitting Pathogenic mtDNA Mutations Females heterozygous for the *PolgA*^MUT^ allele are used to generate germline-transmissible mtDNA mutations and mated to wild-type males. Female offspring, which are wild-type at the *PolgA* locus, are further bred to generate female-derived lines, transmitting the mutated mtDNAs. After establishing maternal lineages to at least the third generation, founder females are sacrificed and their colonic crypts screened. Colonic crypts in founder mice were screened for the presence of mitochondrial dysfunction (some blue crypts on COX/SDH staining). Established mouse lines where the founder showed normal mitochondrial activity (only brown crypts on COX/SDH staining) were discontinued. To identify mtDNA mutations segregating with the mitochondrial dysfunction, the complete mitochondrial genome is sequenced from crypts deficient in mitochondrial function (blue crypts). Using this screening procedure, we identified three distinct lines harboring COX-deficient cells out of the 12 lines. See also Figure S1.

The *PolgA*^MUT/MUT^ genotype causes an approximately equal mutation load in both the germline and somatic tissues (Ross et al., 2013, Stewart et al., 2008), and we therefore estimated that the breeding of *PolgA*^+/MUT^ mice, which have a much lower somatic mtDNA mutation load of ∼2 × 10^−4^ mutations/bp (Ross et al., 2013), would induce approximately three mutations per transmitted mtDNA molecule. We established a breeding strategy to generate female lineages (n = 12 lineages; Figure 1) by first crossing *PolgA*^+/MUT^ males to wild-type C57BL/6N females to obtain *PolgA*^+/MUT^ females with reintroduced wild-type mtDNA. In the subsequent cross (N1), the *PolgA*^+/MUT^ females were crossed with wild-type males to obtain females in the N2 generation that have a wild-type nuclear genome and contain maternally inherited mtDNA mutations. These founder females were further bred to establish maternal lineages to segregate and clonally expand the maternally transmitted mtDNA mutations. To validate that mtDNA mutations indeed had been introduced, we performed complete mtDNA sequencing from eight of these lineages and observed the presence of heteroplasmic mtDNA mutations in all of them from the N3 generation and onward. The pattern of mtDNA mutations in these lineages showed a strong bias against mutations changing the first and second codon positions of protein coding genes, which is consistent with strong purifying selection in the maternal germline (Figure S1; Stewart and Larsson, 2014, Stewart et al., 2008).

### Detection of Clonally Expanded Pathogenic mtDNA Mutations

**Figure 2:**
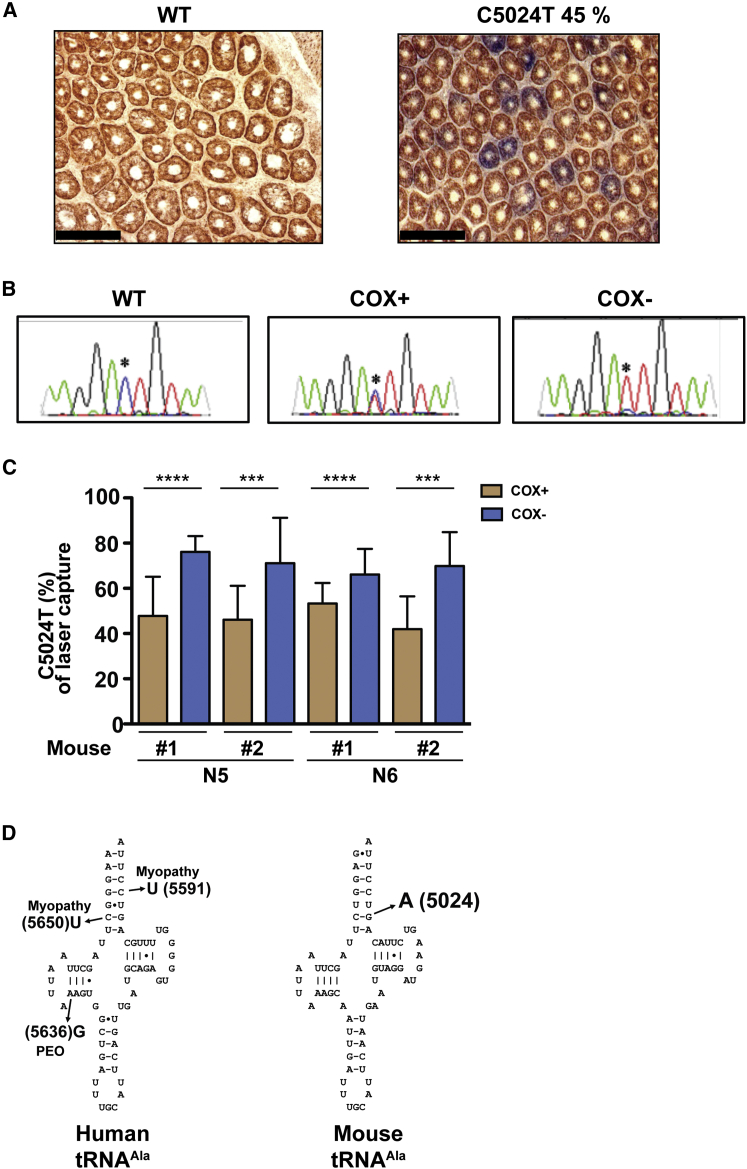
The *tRNA*^*ALA*^ C5024T Mutation Is Identified as a Pathogenic mtDNA Mutation (A) Representative COX/SDH staining of colonic crypts from wild-type mouse (WT) and a mouse that carries the *tRNA*^*ALA*^ C5024T mutation. Black bar represents 100 μm. Crypts, which are brown, have normal COX activity; those that are blue have deficient COX activity. (B) Electropherograms from mtDNA sequences obtained from isolated colonic crypts from WT mice and mice with the C5024T mutation shows the correlation of relative level of C5024T mutation and mitochondrial dysfunction in the colonic crypt. The crypts that are positive for mitochondrial function (COX+) show lower relative levels of the C5024T mutation, and colonic crypts deficient in mitochondrial function (COX−) show higher relative levels of the C5024T mutation. (C) Relative levels of the heteroplasmic C5024T mutation in individually dissected colonic crypts that are either COX positive or COX negative, showing that high levels of the C5024T mutation are present in the COX-negative colonic crypts. Error bars indicate SD. ^∗∗∗^p < 0.001; ^∗∗∗∗^p < 0.0001 (Mann-Whitney *U* test). (D) Clover-leaf representations of the tRNA^ALA^ from humans (left) identifying the positions of known pathogenic mutations and the location of the C5024T mutation in the structure of tRNA^ALA^ from mice (right). See also Figure S2.

The majority of the human mtDNA mutations affect tRNA genes, and they will impair mitochondrial translation if present at high enough levels. Mutations of mtDNA tend to undergo rapid segregation in certain types of stem cells, and somatic mtDNA mutations clonally expand to cause a mosaic respiratory chain deficiency in colonic crypts in both humans (Greaves et al., 2010, Taylor et al., 2003) and mice (Baines et al., 2014). We hypothesized that this clonal expansion phenomenon would be a powerful tool to identify pathogenic mtDNA mutations in our mouse lines and establish their pathogenicity. To this end, we analyzed the founder individual from each of the 12 mouse lines and performed a combined COX and succinate dehydrogenase (SDH) staining of colonic epithelium. With this technique, where respiratory chain deficient cells appear blue and wild-type cells brown, we identified respiratory-chain-deficient cells in the founder mice of 3 of the 12 analyzed mouse lines. Next, we performed laser-capture dissection of individual blue crypts and sequenced mtDNA after PCR amplification. In one of the three lines with COX-deficient colonic crypts (Figure 2A), we found high levels of a heteroplasmic C5024T mutation in the *tRNA*^*ALA*^ gene (Figure 2B). We developed an allele-quantifying pyrosequencing protocol to measure the levels of mutated mtDNA in individual colonic crypts and found a significant correlation between levels of the *tRNA*^*ALA*^ mutation and the occurrence of respiratory chain deficiency (Figure 2C). The identified C5024T mutation in mouse mtDNA disrupts the same base pair in the acceptor stem of tRNA^ALA^ as the pathogenic G5650A mutation found in human patients with a mitochondrial disease syndrome (Figure 2D) (Finnilä et al., 2001, McFarland et al., 2008). The human mutation introduces a second G-U wobble base pair in the acceptor stem, while the mouse mutation generates a C-A mismatch next to a U-U mismatch, which possibly could lead to differences in tRNA^ALA^ stability despite the very similar structural location of the mutations in both species.

In addition to the *tRNA*^*ALA*^ mutation, we found a linked C13715T mutation in the *mt-Nd6* gene, which changes an amino acid (G-to-D substitution at position 119) of ND6. Alignment of sequences from a number of rodents revealed that this site is very poorly conserved and has a poor predicted pathogenicity score (Figure S2A). Generally, mitochondrial tRNA (mt-tRNA) mutations broadly affect mitochondrial translation, with many pathogenic human mutations showing either simultaneous declines in complex I (CI) and complex IV (COX), or more rapid and severe declines in CI relative to COX (Rocha et al., 2015). Examination of the colonic crypts from mice with high levels of the two mutations using antibodies to the CI protein NDUFB8 (Rocha et al., 2015) revealed slightly more CI deficient crypts compared to the number of crypts displaying COX deficiency (Figures S2C–S2E). Such a pattern is consistent with mt-tRNA pathogenicity and did not reveal a strongly enhanced CI defect as one would expect from compounding mutations affecting CI. Mutations in the mitochondrially encoded CI subunits often lead to strong reductions in the amount of CI found and the accumulation of partially assembled CI (Bai and Attardi, 1998, Leman et al., 2015, Leshinsky-Silver et al., 2010, Lim et al., 2016, Lin et al., 2012, Ugalde et al., 2007). Blue native PAGE was performed on mitochondria from animals with high relative levels of the two mutations, and we saw no decrease in the steady-state levels of CI or partially assembled CI (Figure S2E). In-gel CI activity assays did not reveal any biochemical deficiency (Figure S2F). These experiments failed to reveal evidence of a pathogenic role for the *mt-Nd6* mutation, and we thus conclude that this mouse line harbors a single pathogenic mutation in the *tRNA*^*ALA*^ gene that causes the observed respiratory chain deficiency.

### The C5024T Mutation of the *tRNA*^*ALA*^ Gene Is Not Neutrally Transmitted

**Figure 3:**
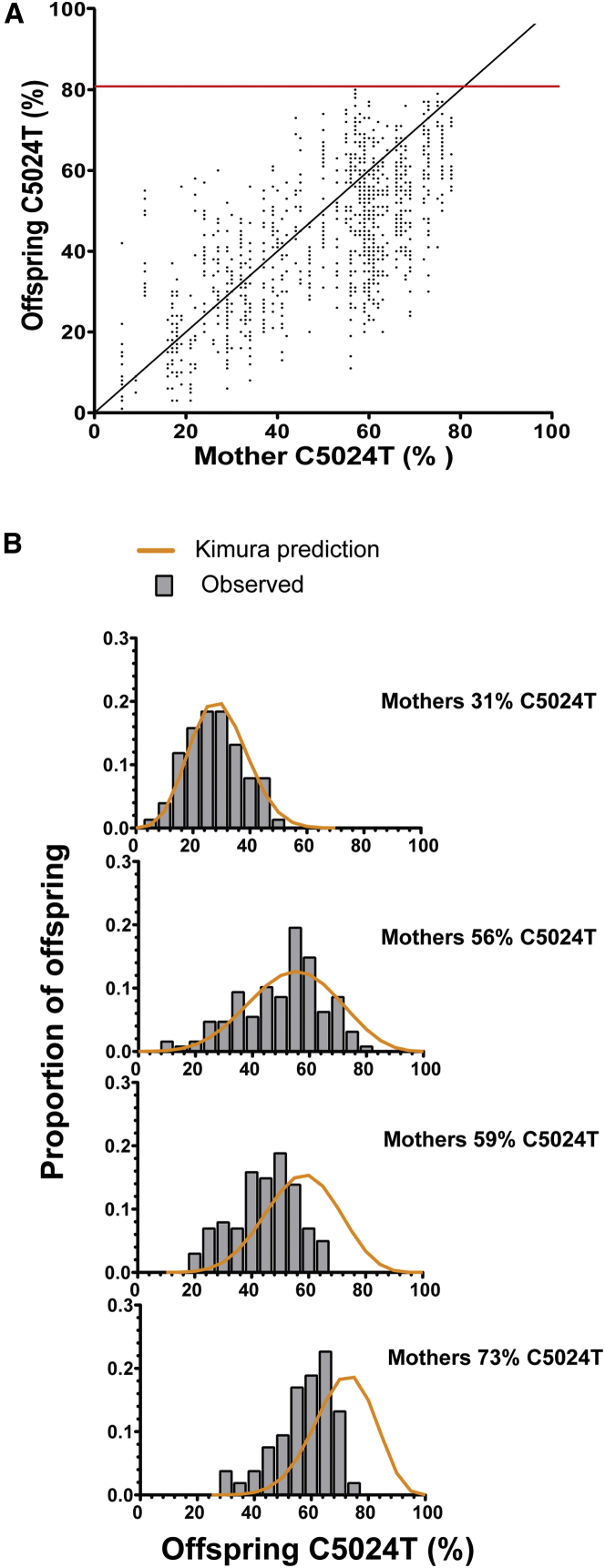
Transmission of the C5024T Mutation in Mice (A) Comparison of the relative levels of the C5024T mutation in 1,105 offspring to heteroplasmic mothers. The red line represents the observed maximum level of the mutation. (B) Four representative tests for neutral segregation of the C5024T mutation using the Kimura distribution (Wonnapinij et al., 2008). Gray bars represent the observed levels of the mutation compared to the expected neutral distribution (orange line). Neutral segregation of the allele was observed until the mother carries 59% of the C5024T mutation, and then the segregation varies from the neutral prediction. All tests are represented in Figure S3.

When breeding mice with the *tRNA*^*ALA*^ mutation, we never observed individuals harboring >80% of the mutation in tail biopsy specimens obtained at the age of 3 weeks (Figure 3A). Using the Kimura model as a null hypothesis for neutral transmission, female mice harboring low levels of the *tRNA*^*ALA*^ mutation (<51%; Figures S3A and S3B) showed a transmission pattern fully consistent with neutral drift (Figure 3B). In contrast, mice with high levels of the mutation (>55%) produced progeny with mtDNA mutation levels that deviated from the neutral drift model (Figure 3B). The number of pups with high mutation levels was much lower than the expected frequency (Figure 3B) despite normal litter sizes (Figure S3C). We have previously demonstrated that tRNA mutations undergo selection in the post-fertilization embryo (Freyer et al., 2012). The behavior of the C5024T mutation implies that this selection occurs in the absence of embryo death, implicating a cellular or organellar phenomenon as the source of this selection. Further work on the mechanisms facilitating this selection is ongoing.

### Reduced Body Mass and Cardiomyopathy in Mutant Mice

**Figure 4:**
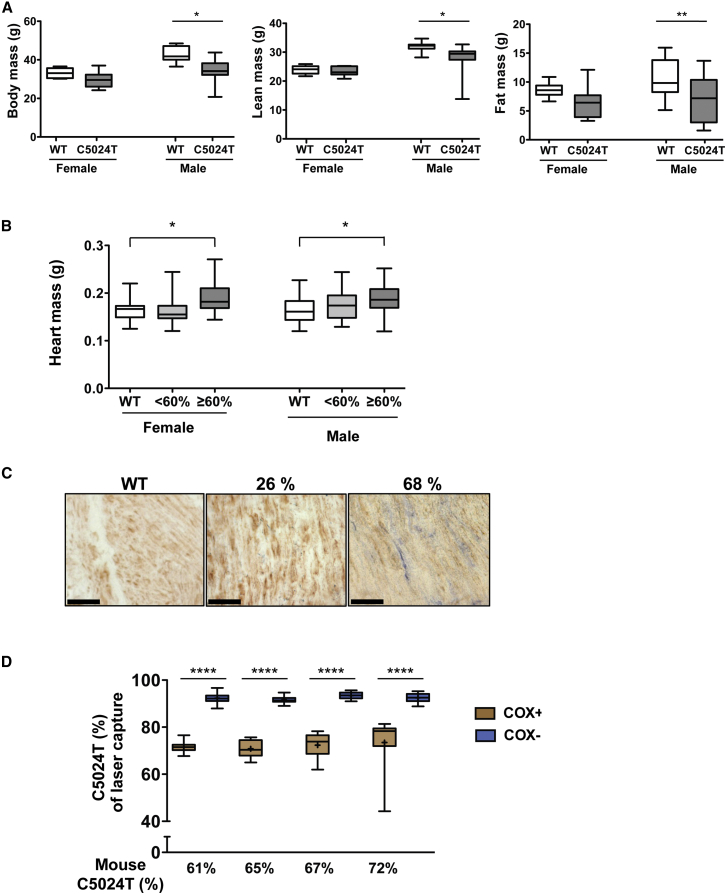
Abnormal Physiology Caused by Mitochondrial Dysfunction in the *tRNA*^*ALA*^ C5024T Mice (A) Male mice carrying the C5024T mutation are smaller than age-matched wild-type animals when comparing body mass, lean mass, and fat content. n = 10–14 for each genotype and gender. Two independent cohorts were analyzed. ^∗^p < 0.05; ^∗∗^p < 0.01 (Dunn’s multiple comparison test). (B) Mice with high relative levels of the C5024T mutation show elevated heart mass, indicative of cardiomyopathy. ^∗^p < 0.05 (Mann-Whitney *U* test). (C) The presence of COX-negative smooth muscle fibers in the colonic smooth muscle of mice with high levels of the C5024T mutation after ∼1 year of age. Black bar represents 50 μm. (D) Relative levels of the C5024T mutation from laser-capture dissected COX-positive and COX-negative smooth muscle fibers, showing tight co-segregation of high levels of the mutation and COX-negative phenotype. n = 15–22 experiments per group. ^∗∗∗∗^p < 0.0001 (Mann-Whitney *U* test). For box-and-whisker plots, bars represent data range, + represents mean, line represents median, and box shows 25^th^–75^th^ percentile of the data. See also Figure S4.

Male mice harboring the *tRNA*^*ALA*^ mutation had reduced total body mass, reduced lean mass and reduced fat content in comparison with control males (Figure 4A), whereas females were unaffected. The heart mass of both female and male mutant mice was increased, especially at high mutation levels (Figure 4B). A variety of tissues were analyzed with COX/SDH enzyme histochemistry to detect respiratory-chain-deficient cells. At the age of 20 weeks, COX deficiency was present in the epithelial cells of colonic crypts, but not in other tissues. At the age of 40 weeks and older, frequent COX deficiency was observed in the smooth muscle surrounding the colon of mice with >60% of the C5024T mutation (Figure 4C) as well as in occasional cardiomyocytes (Figure S4). Laser-capture dissection of colonic smooth muscle cells showed a significant correlation between the levels of the *tRNA*^*ALA*^ mutation and the occurrence of COX deficiency (Figure 4D), again confirming the pathogenic nature of the mutation.

### Selection against High Mutation Levels in Proliferating Tissues

**Figure 5:**
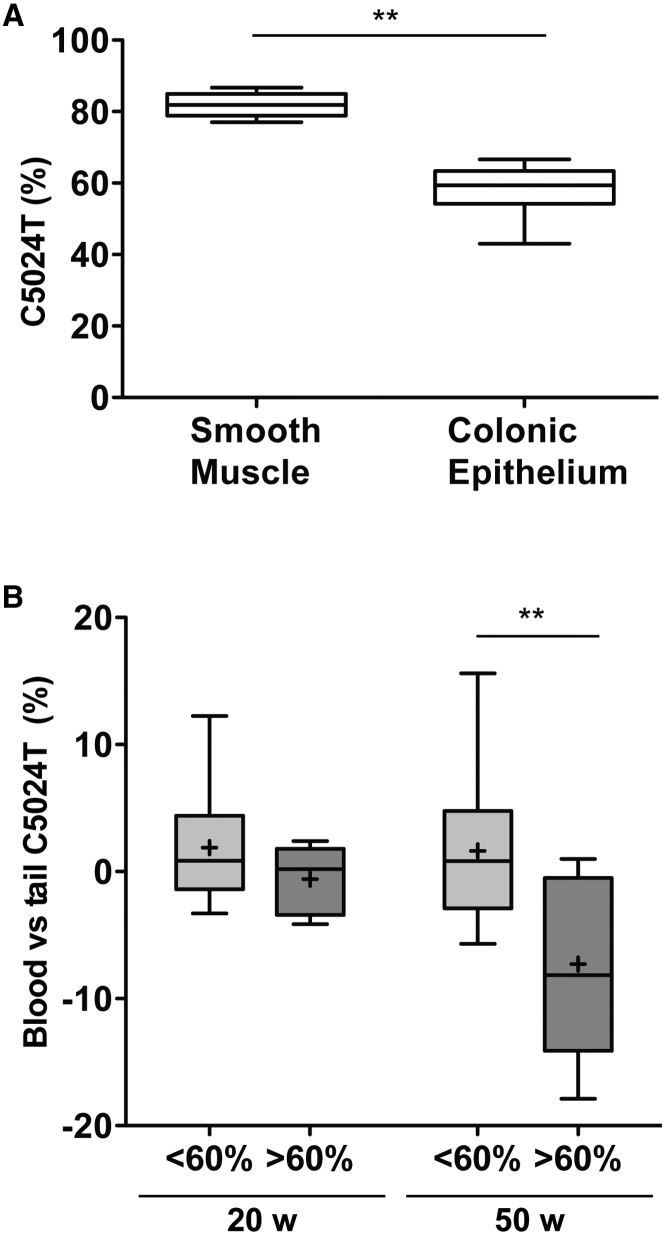
Selective Loss of the C5024T Mutation from Blood and the Colonic Epithelium of Aged Mice (A) Relative levels of the C5024T mutation measured from laser-capture dissected colonic smooth muscle versus colonic epithelium from the same mouse reveals a specific loss of the mutation in the colonic epithelium. n = 8. ^∗∗^p < 0.01 (Wilcoxon matched-pairs signed rank test). (B) Relative levels of the C5024T mutation from blood cells. Animals with high levels of the mutation (>60% in other tissues) show decreased mutation levels in blood at 50 weeks of age. ^∗∗^p < 0.01 (Mann-Whitney *U* test). For box-and-whisker plots, bars represent data range, + represents mean, line represents median, and box shows 25^th^–75^th^ percentile of the data. See also Figure S5.

The levels of mutated mtDNA were similar in all analyzed tissues in young animals (age ∼20 weeks) and reflected the levels in tail biopsy specimens obtained at weaning (age ∼3 weeks) (Figure S5A). In contrast, the mutation levels were decreased in the highly proliferative colonic epithelium in comparison with the surrounding smooth muscle in older mice (age >40 weeks) (Figure 5A). Interestingly, a similar decrease of mutation levels was seen in peripheral blood of older mutant animals (Figure 5B). Taken together, these findings show that tissues with high proliferation can select against high levels of the *tRNA*^*ALA*^ mutation, whereas the levels remain constant over time in less proliferative tissues. Interestingly, this type of selection manifested as low mutation levels in peripheral blood is also seen in some human mtDNA mutation disease syndromes, such as CPEO (Larsson and Clayton, 1995) and the A3243G MELAS mutation (Ciafaloni et al., 1991), whereas others show a good correlation between mutation levels in peripheral blood and skeletal muscle, such as the G8344A mutation, which causes MERRF syndrome (Larsson et al., 1992).

### The *tRNA*^*ALA*^ Mutation Impairs Mitochondrial Translation

**Figure 6:**
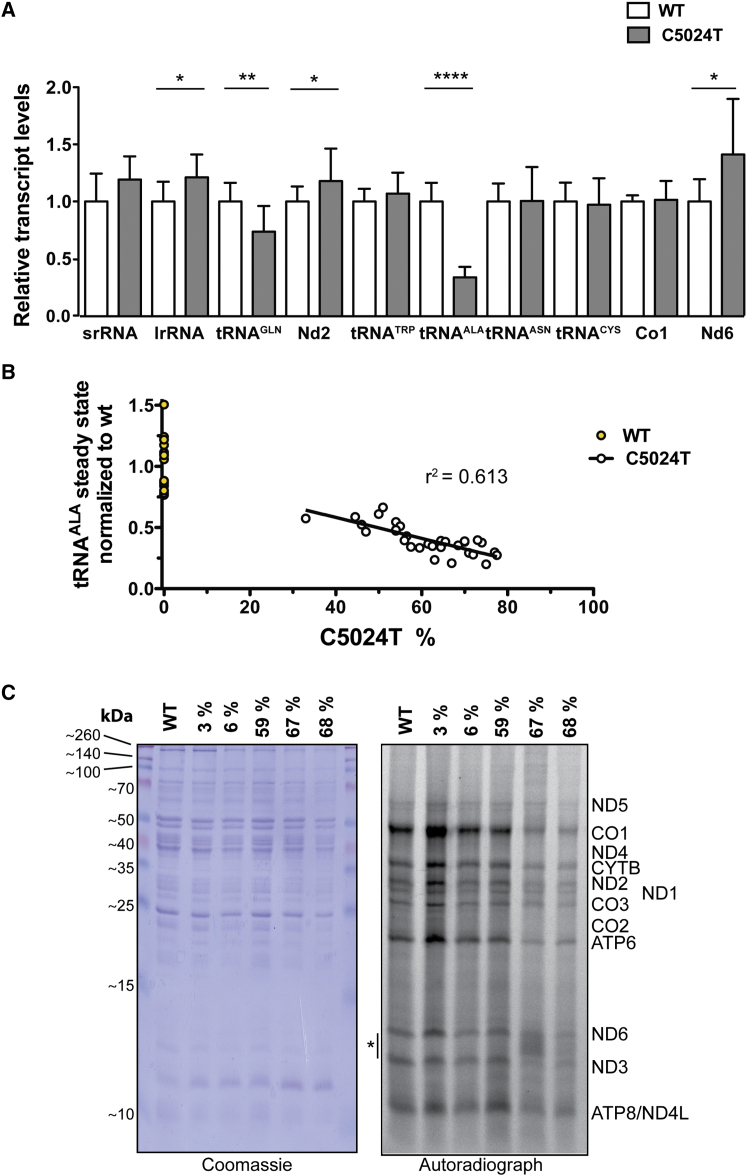
*tRNA*^*ALA*^ C5024T Leads to Depletion of tRNA^ALA^ and Deficiency in Mitochondrial Translation (A) Northern blot analyses of various mitochondrial tRNA, rRNA and mRNA transcripts from heart reveals strong depletion of the steady-state levels of tRNA^ALA^, and a mild increase in steady-state levels of some other mitochondrial transcripts. Data are pooled from three independent experiments. WT, n = 12; C5024T, n = 19 (mean age, 65 weeks; C5024T 44%–77%). Error bars represent SD. ^∗^p < 0.05; ^∗∗^p < 0.01; ^∗∗∗∗^p < 0.0001 (Mann-Whitney *U* test). (B) Steady-state levels of tRNA^ALA^ in comparison with the relative levels of mtDNA with the C5024T mutation in heart. n = 31. ^∗^p < 0.0001 (linear regression). (C) In organello translation of mitochondria isolated from heart reveal decreased translation capacity in tissues harboring high levels of the mutation and the occasional appearance of low-molecular-weight aberrant translation products (^∗^) consistent with prematurely terminated or stalled translation. As a loading control, Coomassie blue staining of proteins after SDS-PAGE is shown. See also Figure S6.

We analyzed the steady-state levels of different mtDNA-encoded RNAs and found a substantial decrease in the tRNA^ALA^ levels in the mutant animals (Figures 6A and S6). The other analyzed tRNAs were present at either normal (tRNA^CYS^, tRNA^ASN^, and tRNA^TRP^) or slightly decreased levels (tRNA^GLN^; Figure 6A). The levels of the *srRNA* and *lrRNA* were slightly elevated, whereas the steady-state levels of the analyzed mRNAs were unaltered (Co1) or slightly elevated (Nd2 and Nd6; Figure 6A). We found low total levels of tRNA^ALA^ in animals with high levels of mutated mtDNA (Figure 6B), consistent with the idea that the mutation impairs the stability of tRNA^ALA^. We proceeded to investigate how the reduced levels of tRNA^ALA^ affected mitochondrial translation (Figure 6C). The in organello protein synthesis was decreased in tissues containing high amounts of mutated mtDNA, showing that the *tRNA*^*ALA*^ mutation indeed impairs mitochondrial translation (Figure 6C), and is consistent with observations in human cell lines and tissues that a certain threshold of mutated mtDNA is needed to impair mitochondrial protein synthesis (Hayashi et al., 1991, Larsson et al., 1992). The amino acid alanine is present in all mtDNA-encoded proteins and represents ∼5% of all amino acids in those proteins, which explains the observed general decrease of mitochondrial translation in animals with high levels of the *tRNA*^*ALA*^ mutation. In mitochondria with high levels of the *tRNA*^*ALA*^ mutation and, consequently, markedly impaired overall mitochondrial translation, we occasionally observed aberrant translation products of low molecular weight (Figure 6C) consistent with translational stalling or pre-mature termination of translation.

## Discussion

We describe here a technically simple phenotype-driven approach to create mouse models for mitochondrial disease. The protocol is based on breeding *PolgA*^*+/*MUT^ females to derive founder mice with a wild-type nuclear background followed by identification of the pathogenic mtDNA mutations by analysis of respiratory chain deficiency in colonic crypts. The founder mice are only sacrificed for analysis of colon after the maternal lines have been established from them. Pathogenic mtDNA mutations frequently undergo clonal expansion in tissues with high cell proliferation (Baines et al., 2014, Greaves et al., 2010, Taylor et al., 2003) and we therefore utilized COX/SDH histochemistry to detect mosaic COX deficiency in colonic epithelium. Laser-capture dissection and mtDNA sequencing of single crypts was then used to identify the mutation and its pathogenicity was established by comparing the mutation levels in crypts with normal or deficient COX activity. The unusually rapid clonal expansion of mtDNA mutations in colonic crypts allows identification of pathogenic mutations in founder mice, despite the fact that they have low levels of the mutation. In fact, the mutations are identified before the onset of any obvious disease phenotypes and even before the mutations are detectable by standard Sanger sequencing of tissue samples. Although the current study used only COX staining to identify candidate mutations, immunohistochemical staining of colonic crypts with anti-NDUFB8 antibodies in concert with the COX/SDH staining could allow the detection of mice with isolated CI deficiencies in future screens. In contrast, an alternate strategy based on large-scale phenotyping of mouse lines to detect heart, skeletal muscle, or CNS dysfunction is very difficult and labor intensive, because pathogenic mtDNA mutations are typically heteroplasmic and cause pleiotropic symptoms that may vary with age (Larsson and Clayton, 1995). Furthermore, a purely sequence-driven approach to identify pathogenic mtDNA mutations is also problematic, because accurate prediction of which mutations will be pathogenic in the mouse is quite challenging with only human clinical data as a guide. The approach we present here also has the advantage that it does not involve labor-intensive manipulation of ESCs or mouse embryos but instead is technically simple and based on breeding mutant mice. Furthermore, only pathogenic mutations that have already been maternally transmitted in the germline are present in the founder mice. This very much increases the likelihood that the identified mtDNA mutations are tolerated in the germline and can be stably transmitted in the maternal lines derived from the founder mice. It should be noted that there are many examples where pathogenic mtDNA mutations found in cell lines or somatic tissues will not be inherited once introduced into mice (Fan et al., 2008, Levy et al., 1999), because they are subject to strong purifying selection in the maternal germline (Stewart and Larsson, 2014).

To show the feasibility of our approach, we report the generation of a mouse line with a pathogenic heteroplasmic C5024T mutation in the *tRNA*^*ALA*^ gene of mtDNA, which recapitulates important aspects of human mitochondrial disease. Importantly, many pathogenic human mtDNA mutations are heteroplasmic and will only cause respiratory chain deficiency if present above a certain threshold. Patients with mitochondrial diseases often harbor wild-type genomes, and interventions to target the mutant mtDNA should therefore be a viable strategy to restore respiratory chain function and improve the clinical condition. Mitochondrial targeting of a restriction enzyme provided the first proof of concept that it is possible to selectively target one of the genotypes in mice with heteroplasmic mtDNA mutations (Bayona-Bafaluy et al., 2005). Unfortunately, because most pathogenic mutations do not alter a single restriction site in mtDNA, mitochondrial targeting of restriction enzymes cannot be used as a general approach. Fortunately, there has been substantial recent progress in developing mitochondrially targeted transcription activator-like effector nucleases (TALENs) or zinc-finger nucleases that specifically cut mutant mtDNA to shift the ratio of mutant to wild-type mtDNA in human cell lines and oocytes (Bacman et al., 2013, Gammage et al., 2014, Minczuk et al., 2006, Reddy et al., 2015). The *tRNA*^*ALA*^ mutant mice we describe here will be a very important tool for development of protocols for in vivo targeting of mutant mtDNA with the aim of restoring respiratory chain function and improving phenotypes. Furthermore, the straightforward protocol we have developed will allow the generation of a variety of mouse models with different types of pathogenic mtDNA mutations, which will be invaluable tools to sort out the puzzling enigma of genotype-phenotype correlations in mitochondrial disease.

## Experimental Procedures

### Animal Husbandry

The mouse strain used in all experiments was the C57Bl/6NCrl mouse (Charles River Laboratories, Germany strain code 027). Mice were housed in a 12-hr light/dark cycle at 21°C and fed ad libitum on a standard mouse food (ssniff M-H Low-Phytoestrogen) or an enhanced diet when breeding or newly weaned mice (ssniff M-Z Low-Phytoestrogen) by Ssniff Spezialdiaeten GmbH. Detailed animal husbandry protocols are available upon request.

All animal work was performed in accordance to recommendations and guidelines of the Federation of European Laboratory Animal Science Associations (FELASA). All experiments were approved and permitted by the Landesamt für Natur, Umwelt und Verbraucherschutz Nordrhein-Westfalen in accordance with German and European Union regulations.

### Generation of Mice with mtDNA Mutations

The mouse colony containing the proofreading-deficient variant of the mtDNA polymerase (Pol^D257A^; referred to as *PolgA*^MUT^) (Trifunovic et al., 2004) is maintained by continuous backcrossing of males heterozygous for the mutant allele to wild-type C57Bl/6NCrl females from a pure wild-type colony within the facility to avoid the accumulation of excess mtDNA mutations (Ross et al., 2013). To generate the lines, females heterozygous for the *PolgA*^MUT^ allele were selected from the breedings described above and crossed with wild-type C57Bl/6NCrl males. Wild-type females from these crosses were then selected to propagate the resulting mtDNA mutations by continuous backcrossing with wild-type C57Bl/6NCrl males.

### Tissue Preparation for Histological Analysis

Mice were sacrificed with CO_2_ and cervical dislocation. Heart and colon tissues were removed and washed with PBS to remove remaining blood or feces. Tissues that were used for histological staining were frozen in isopentane (15 s) that was previously cooled to −160°C in liquid nitrogen. Tissues used in other analyses were snap frozen in liquid nitrogen. Sections were cut (10 μm colon, 7 μm heart) with an OFT 5000 cryostat (Bright). The sections used for histological staining were cut on to glass colorcoat adhesion slides (CellPath) and the sections for laser-capture dissection (15 μm) on to polyethylenenaphthalate (PEN) slides (Leica Microsystems). Both sections were stored in −80°C prior to further analysis.

### Dual COX/SDH Enzyme Histochemistry

Sections were incubated in 50 μl COX staining medium (100 μM cytochrome *c*, 4 mM diaminobenzidine tetrahydrochloride, 20 μg/ml catalase, and 0.2 M phosphate buffer [pH 7.0]) at 37°C for 25 min for colon and for heart sections 60 min at 37°C, followed by three 5-min washes with PBS. Next, the sections were incubated with 50 μl SDH solution (130 mM sodium succinate, 200 μM phenazinemethosulphate, 1 mM sodium azide, 1.5 mM nitroblue tetrazolium, and 0.2 M phosphate buffer [pH 7.0]) and incubated at 37°C for 35 min for colon and at room temperature for 30 min for heart, followed by three 5-min washes with PBS and dehydration through graded ethanol series (70%, 95%, and 2× 100%), clearing in Histoclear (National Diagnostics), and mounting in DPX. The sections for laser-capture dissection (15 μm) that were on PEN slides were similarly exposed to COX/SDH staining, dehydrated though graded ethanol series, and air-dried for 90 min.

### mtDNA Sequence Analysis

Detailed protocols describing DNA sequencing are published elsewhere (Baines et al., 2014, Ross et al., 2013, Stewart et al., 2008). Briefly, 30 overlapping M13-tagged primer pairs were used to amplify the mtDNA. Sequencing reactions were carried out using the M13 primer tags with Big Dye 3.1-based sequencing chemistry and purification using the BigDye Xterminator cleanup kit. Sequences were resolved on an ABI 3730 DNA Analyzer, using 50-cm capillary arrays and long sequencing run protocols. mtDNA sequences were assembled using SeqScape Version 2.7. Mixed-base calls used a >20% threshold to detect heteroplasmic mtDNA mutations, which were manually confirmed for each mutation call. Alignment issues due to the linear representation of the circular mtDNA sequencer were overcome by duplication of the first 212 bp of the mtDNA sequence on the reference genome’s 3′ end.

### Quantification of C5024T Mutation Load

Two methods were used during the course of this study to quantify the relative levels of the mutant and wild-type mtDNAs. The first method involved a modified RFLP analysis (similar to Freyer et al., 2012). The second method involved Allelic Quantification analysis using a PyroMark Q24 pyrosequencer (QIAGEN). Details of the assays can be found in Supplemental Experimental Procedures.

### Laser-Capture Microdissection

Laser-capture-microdissected tissues were cut to 15 μm thickness, mounted on PEN slides (Leica Microsystems), and air-dried at room temperature for 1 hr. Slides were stored in sealed slide mailers at −80°C until use. Microdissection was carried out on a Leica Microsystems LMD7000 laser microdissection microscope, sorting a single colonic crypt section or five smooth muscle fibers into single tubes for further analysis. Captured colonic crypts or muscle fiber sections were settled to the bottom of the tube by centrifugation at 7,000 rcf for 10 min. DNA was extracted in 10 μl lysis buffer (50 mM Tris-HCl [pH 8.5], 1% Tween-20, and 20 mg/ml proteinase K) for at least 2 hr and 55°C, with a heat-inactivation step at 95°C for 10 min (Taylor et al., 2003). The extraction was used directly in PCR reactions for mutation level quantification or diluted in 30 μl water for PCR and sequencing.

### Complex I Immunohistochemisty

Colons were fixed in 4% paraformaldehyde (PFA) overnight at room temperature. The formalin was then removed and the intestines washed in 70% ethanol before standard processing for paraffin embedding. 4-μm sections were cut and incubated at 37°C overnight to ensure adherence to the slides. Sections were de-paraffinized in two changes of Histoclear and rehydrated in a graded EtOH series. Antigen retrieval was carried out by pressure cooking in 1 mM EDTA (pH 8.0) for 20 min. Endogenous peroxidase activity was blocked by the addition of 0.3% H_2_O_2_ to the 95% EtOH during the rehydration step. Sections were then incubated in 10% normal goat serum for 1 hr at room temperature. Endogenous biotin was blocked using an avidin/biotin blocking kit (Vector Laboratories). Sections were then incubated overnight at 4°C in mouse anti-NDUFB8 (Abcam) diluted 1:100 in 10% normal goat serum in Tris-buffered saline. Following washing in Tris-buffered saline plus Tween 20 buffer, sections were incubated goat anti-IgG1-biotin (Jackson ImmunoResearch Laboratories) for 2 hr at 4°C, followed by incubation with the VECTASTAIN Elite ABC (Vector Laboratories) as instructed by the manufacturer. NDUFB8 expression was assessed in four sections from three mice. Sections were taken at 100-μm intervals.

### Test of Neutral Segregation in the Female Germline

The segregation of the allele was tested against a neutral model using the Kimura distribution (Wonnapinij et al., 2008). The Kimura003.c code was downloaded, and the initial population mutation proportion in the mothers, sample size, and variance were modified in the code before compiling for each run. Large sample numbers are required for robust analysis, so multiple females with ±4% of the C5024T mutation and their offspring were grouped together for each analysis. The weighted mean of the C5024T mutation levels in the 3-week-old tail or earclip biopsy specimens from the mothers was used to define p. *Var* was calculated from the population of pup 3-week-old tail or earclip biopsy measurements. The result of the statistical test for conforming to the neutral distribution was retrieved from the monte_carlo1000.txt file output from each analysis.

### Body Composition Measurements

Body fat and lean content were measured in vivo by nuclear magnetic resonance using a minispec LF50H (Bruker) at the Phenotyping Core Facility of MPI for Biology of Aging.

### RNA Isolation and Northern Blot Analysis

Total RNA was isolated from snap frozen heart tissues with TRIzol (Ambion) following the manufacturer’s standard protocol. The extracted RNA was quantified with the Nanodrop and resolved on formaldehyde-agarose gel followed by a transfer onto Hybond-N membrane (GE Healthcare). Transcripts of interest were detected with non-radioactive method essentially as previously described (Davies et al., 2012). Briefly, the membrane was probed with mouse-specific biotin-labeled oligonucleotides by overnight incubation in hybridization buffer (5× saline sodium citrate [SSC], 20 mM Na_2_HPO_4_, 7% SDS, 0.5× RNA secure [Ambion], and 100 μg/ml heparin) at 50°C followed by washing and signal detection with IRDye 800CW dye-labeled streptavidin (dilution 1:5000 in TBS, 0.05% tween-20) in a LI-COR Biosciences imaging system.

### Blue Native PAGE Electrophoresis

Isolation of mitochondria from mouse hearts was performed by differential centrifugation (Mourier et al., 2014). 100 μg mitochondria was lysed with 50 μl solubilization buffer (1% [w/v] digitonin [Calbiochem], 20 mM Tris-HCl [pH 7.4], 0,1 mM EDTA, 50 mM NaCl, and 10% [v/v] glycerol), and blue native PAGE (BN-PAGE) was performed as previously described (Mourier et al., 2014).

### Western Blot Analysis

Proteins were separated by BN-PAGE and transferred to polyvinilidene difluoride (PVDF) membrane by semi-dry transfer. Polyclonal NDUFV2 (1:1,000, Sigma) was used for detection of Complex I and chemiluminescence detection was performed by using Amersham ECL Prime western blotting detection reagent. To assure equal loading, BN-PAGE gels were stained with Coomassie solution (Phast Gel Blue R-350, GE Healthcare).

### In Gel Activity Assay

To assess the activity of CI, BN-PAGE gels were incubated in 2 mM Tris-HCl (pH 7.4), 0.1 mg/ml NADH (Roche), and 2.5 mg/ml iodonitrozolium (Sigma) for 30 min at room temperature. The reaction was stopped by transferring the gels to 2 mM Tris-HCl (pH 7.4).

### In Organello Translation

Mitochondria were extracted from freshly isolated hearts by differential centrifugation. The purified mitochondria were incubated at 37°C in translation buffer (100 mM mannitol, 10 mM sodium succinate, 80 mM KCl, 5 mM MgCl_2_, 1 mM KH_2_PO_4_, 25 mM HEPES, 200 mM ATP, 5 mM GTP, 200 μM creatine phosphate, 6 mM creatine kinase, 100 μg/ml emetine, and 200 μg/ml cycloheximide with 6 μg/ml of every amino acid except methionine) and 115 μCi/ml 35S-labeled methionine for 60 min. Next, the mitochondria were washed in translation buffer and resuspended into 2× Laemmli buffer (125 mM Tris [pH 6.8], 4% SDS, 20% glycerol, 1.4 M 2-mercapto-ethanol, and 0.025% bromophenol blue.) The mitochondrial proteins were resolved on a 17% SDS-PAGE gel and analyzed by autoradiography. Equal loading of the mitochondria was controlled by Coomassie staining.

### DNA Extraction from Coagulated Blood

Following cervical dislocation, total blood was removed from the thoracic cavity of the mouse and allowed to coagulate at room temperature for 2 hr. Serum was then separated from the blood with 2,000 × *g* centrifugation for 20 min at room temperature. After storage at −80°C, DNA was extracted from the coagulated blood clot with Nucleospin 96 well blood quick pure kit (MN) following the manufacturer’s recommendations. The extracted DNA was used to detect C5024T mutation levels with pyrosequencing.

### Statistical Analysis

All statistical analyses were performed and graphs were drawn with GraphPad Prism v6 software. α = 0.05 was used for all tests, with multiple-testing corrections where appropriate. Typically, non-parametric tests were used for the data analyzed. Exact tests used are referred to in the figure legends.

## Author Contributions

Conceptualization, N.-G.L., L.C.G., and J.B.S.; Methodology, H.L.B., L.C.G., and J.B.S.; Investigation, J.H.K.K., H.L.B., A.B., M.-L.S., C.F., A.M., C.S., and J.B.S.; Validation, R.F.; Writing – Original Draft, J.H.K.K., N.-G.L., and J.B.S.; Writing – Review and Editing, J.H.K.K., H.L.B., A.B., M.-L.S., C.F., A.M., C.S., R.F., N.-G.L., L.C.G., and J.B.S.; Supervision, N.-G.L., L.C.G., and J.B.S.

## Supplemental Information

Document S1. Supplemental Experimental Procedures and Figures S1–S6 Document S1. Supplemental Experimental Procedures and Figures S1–S6

Document S2. Article plus Supplemental Information Document S2. Article plus Supplemental Information
