# Supplementary material for: A Phenotype-Driven Approach to Generate Mouse Models with Pathogenic mtDNA Mutations Causing Mitochondrial Disease
Source: Cell Rep. 2016 Sep 13;16(11):2980–90. doi: 10.1016/j.celrep.2016.08.037 (PMC5039181; doi:10.1016/j.celrep.2016.08.037)
Supplement: Document S1. Supplemental Experimental Procedures and Figures S1–S6 [file mmc1.pdf]

Cell Reports, Volume *16*

Supplemental Information

A Phenotype-Driven Approach to Generate Mouse Models with Pathogenic mtDNA Mutations Causing Mitochondrial Disease

Johanna H.K. Kauppila, Holly L. Baines, Ana Bratic, Marie-Lune Simard, Christoph Freyer, Arnaud Mourier, Craig Stamp, Roberta Filograna, Nils-Göran Larsson, Laura C. Greaves, and James B. Stewart

Supplemental Information

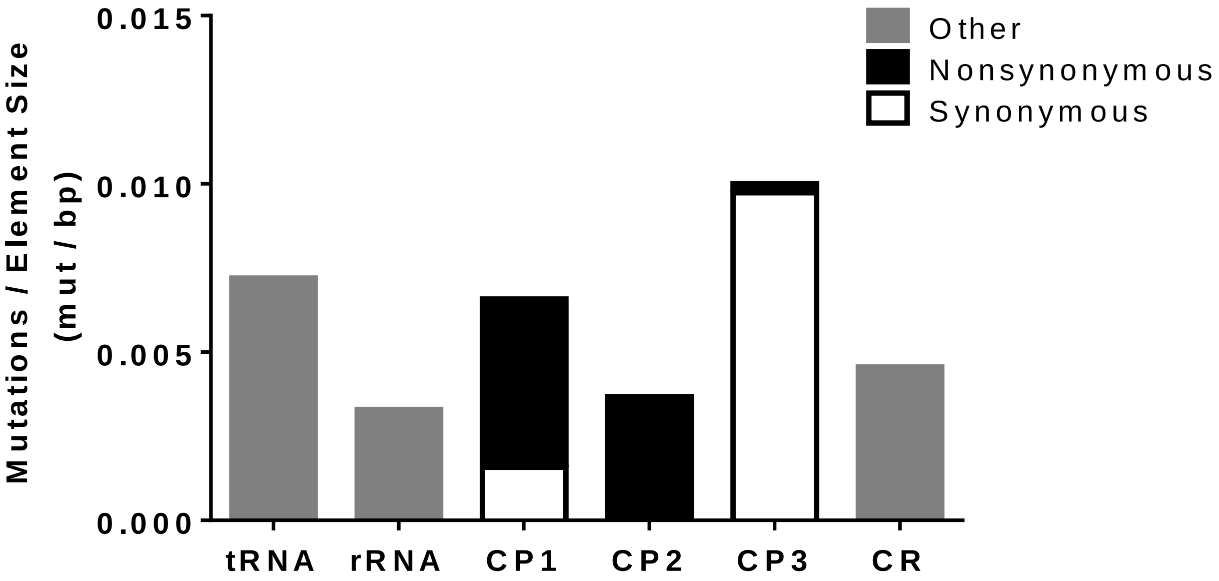

Supplemental Figure S1. Related to Figure 1.

The distribution of mutations reveals purifying selection in *PolgA*$^{+/MUT}$ derived lineages. The mtDNA mutations observed in these lineages show a reduction in amino-acid changing mutations in first and second codon positions (CP1 & CP2) relative to silent mutation in first or third codon (CP3) positions being transmitted through the female germline, as observed in *PolgA*$^{MUT/MUT}$ derived lineages [22]. The frequency of tRNA and Control Region (CR) mutations appears similar to those on the *PolgA*$^{MUT/MUT}$, though rRNA mutations may be less frequent than reported in that study. The total distribution of mutations observed is not significantly different from the *PolgA*$^{MUT/MUT}$ study (Chi-squared test, p=0.4316).

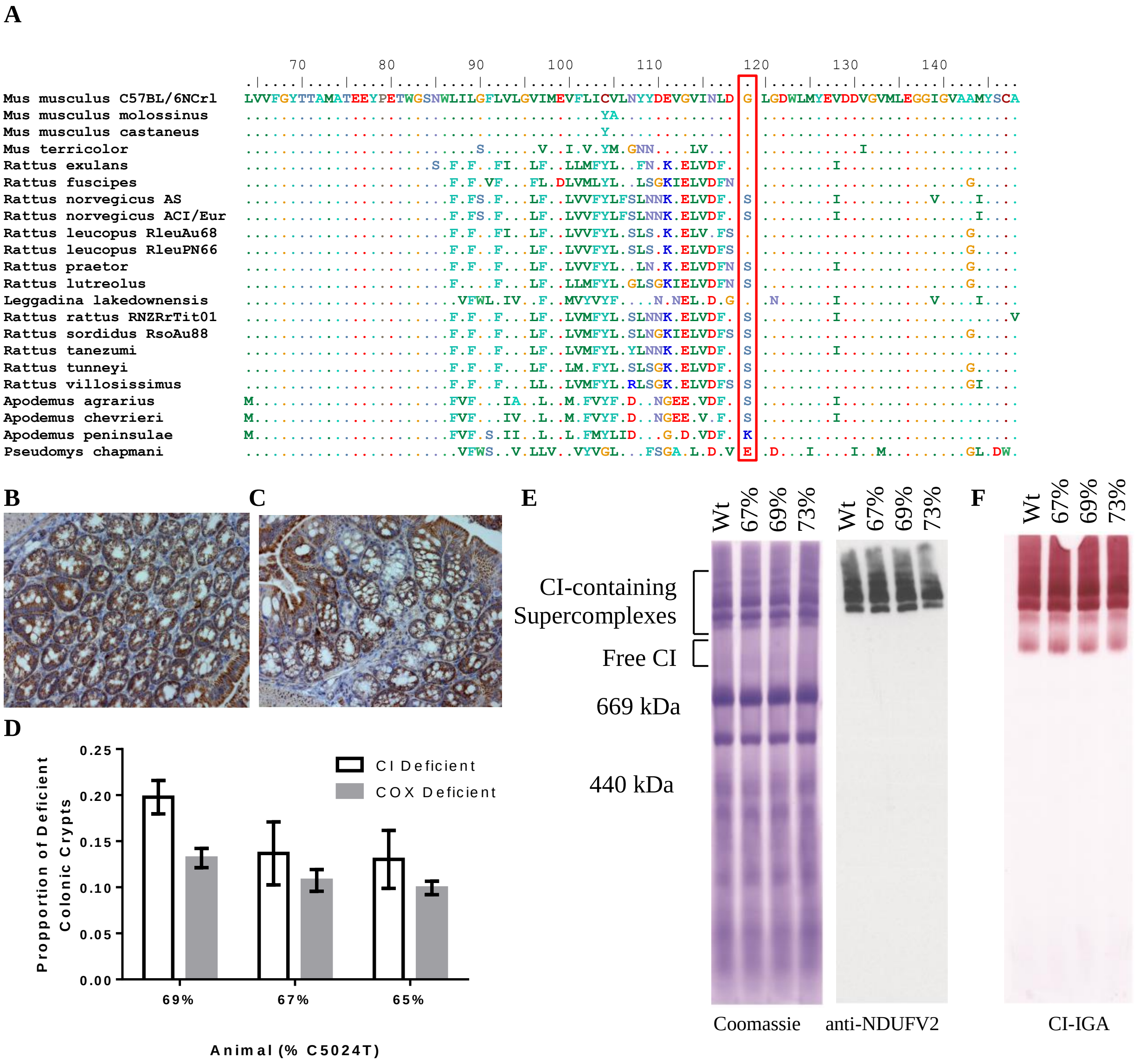

Supplemental Figure S2. Related to Figure 2.

(A) The C5024T mutation is linked to a non-synonymous C13715T mutation in *mt-Nd6*. Of 240 rodent mitochondrial genomes obtained from GenBank, the 22 variants of the ND6 amino acid sequence in the aligned region were identified. The C13715T (G119D) mutation is highlighted by the red box. This mutation received a low MUTPRED general pathogenicity score of 0.473 (http://mutpred.mutdb.org/), and is found in a poorly conserved portion of the protein, arguing against a pathogenic role of this mutation

(B) Immunohistochemical CI staining of a wildtype animal's colonic crypts with anti-NDUFB8 antibodies.

(C) The same staining on an animal with 67% C5024T-C13715T showing light blue CI deficient crypts.

(D) The proportion of COX and CI deficient colonic crypts for the same individuals. Mean of four slides counted, error bars = SD.

(E) Representative coomassie stained Blue Native PAGE-Gel (BNPG) and anti-NDUFV2 western blot of a BNPG reveal no decrease in CI or in CI supercomplex assembly in animals with high levels of the C5024T/C13715T mtDNA.

(F) In-gel activity assay of a BNPG showing normal CI activity.

A

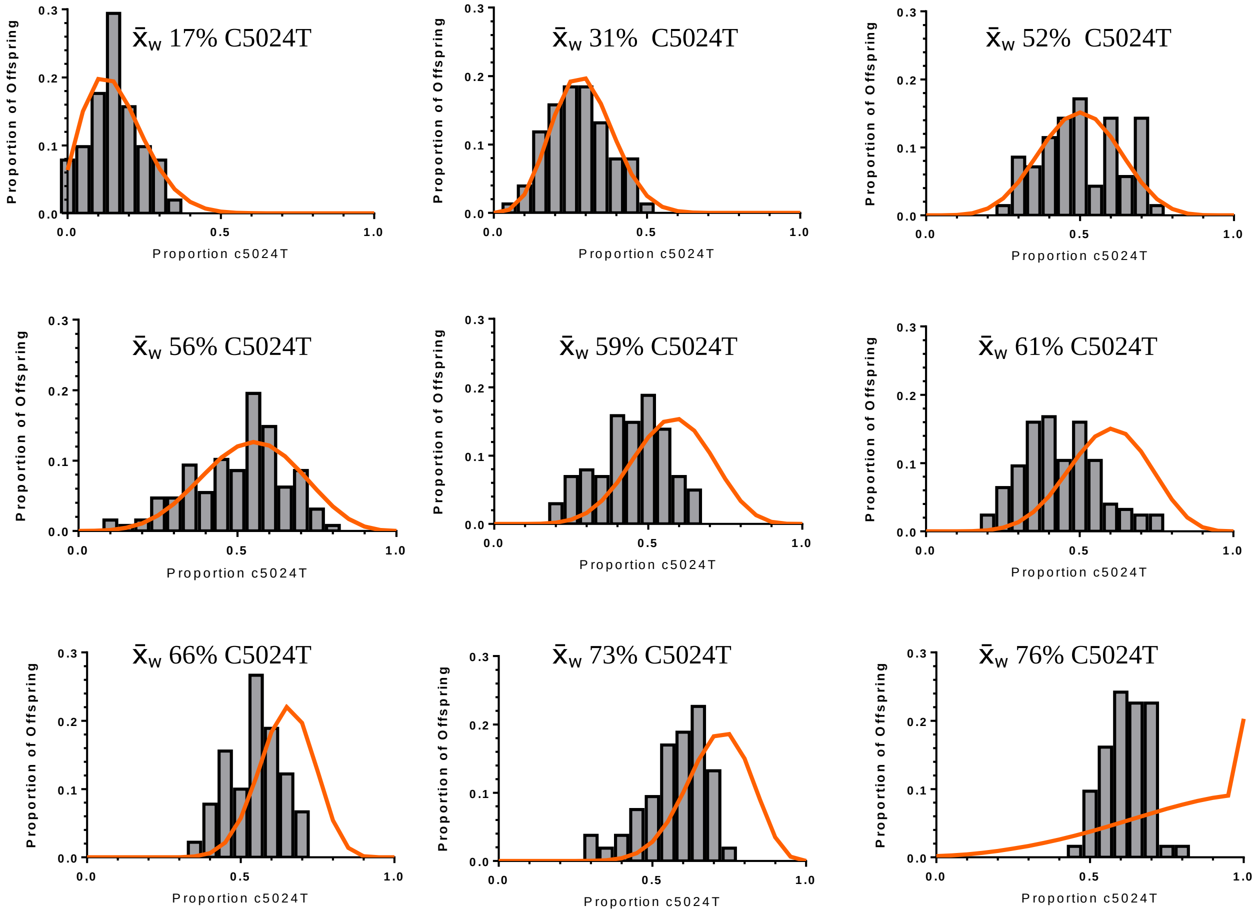

B

Weighted Mean of Mothers ($\bar{x}_w$)	Number of Data Points	Sample Variance	p value
**0.174**	56	0.00978	0.838
**0.308**	81	0.00940	0.538
**0.515**	75	0.01608	0.520
**0.559**	75	0.02251	0.033
**0.590**	106	0.01550	< 0.000001
**0.608**	130	0.01628	< 0.000001
**0.663**	95	0.00774	< 0.000001
**0.725**	58	0.01057	< 0.000001
**0.756**	67	0.00493	< 0.000001

C

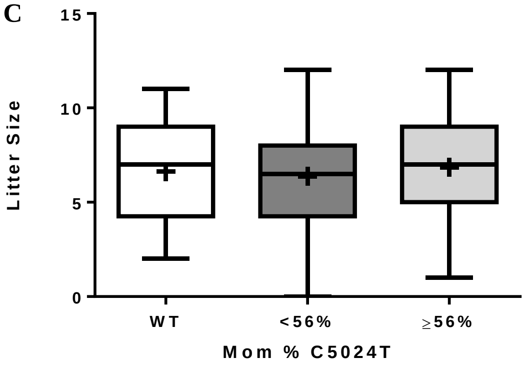

Supplemental Figure S3. Related to Figure 3.

(A) Tests of variation from the Kimura Distribution for pups born to mice with varying levels of the *tRNA*^{ALA} C5024T mutation. Grey bars represent the observed levels of the mutation compared to the expected neutral distribution (orange line). Weighted mean of the mothers ($\bar{x}_w$) is displayed for each panel.
(B) Variables and summary statistics used in test of germline neutral segregation of the *tRNA*^{ALA} C5024T mutation.
(C) Litter sizes from female mice carrying ≥56% of the *tRNA*^{ALA} C5024T mutation (N=102 litters) were not different from those of mice with <56% of the (N=80) or age-matched wildtype females of the same nuclear genetic background (N=84) (Not significant, 1 way ANOVA, with Dunn's correction multiple comparisons between all groups. Bars = data range, + = mean, line = median, box = 25-75th percentile of the data, $\bar{x}_w$ = weighted mean of the mothers).

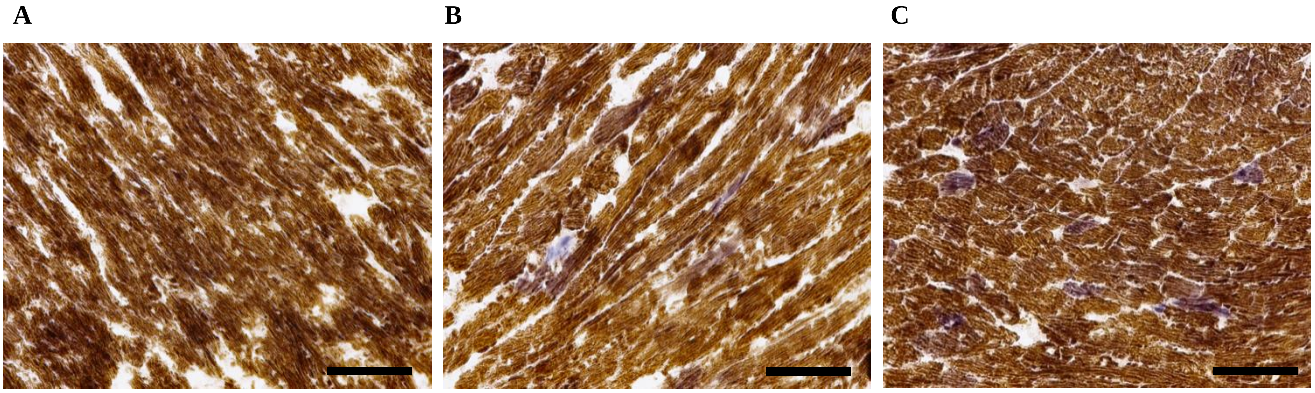

Supplemental Figure S4. Related to Figure 4.
Rare COX negative cardiomyocytes are detected in mice with high relative levels of *tRNA*^{ALA} C5024T mutation. Representative COX-SDH staining of heart tissue from **(A)** wild-type mouse and **(B,C)** a mouse that carries a relative level of 80% for the *tRNA*^{ALA} C5024T mutation. Brown staining represents cells with normal mitochondrial function, while blue staining reveals cells with mitochondrial dysfunction (in B and C). Black bar represents 100 μm.

A

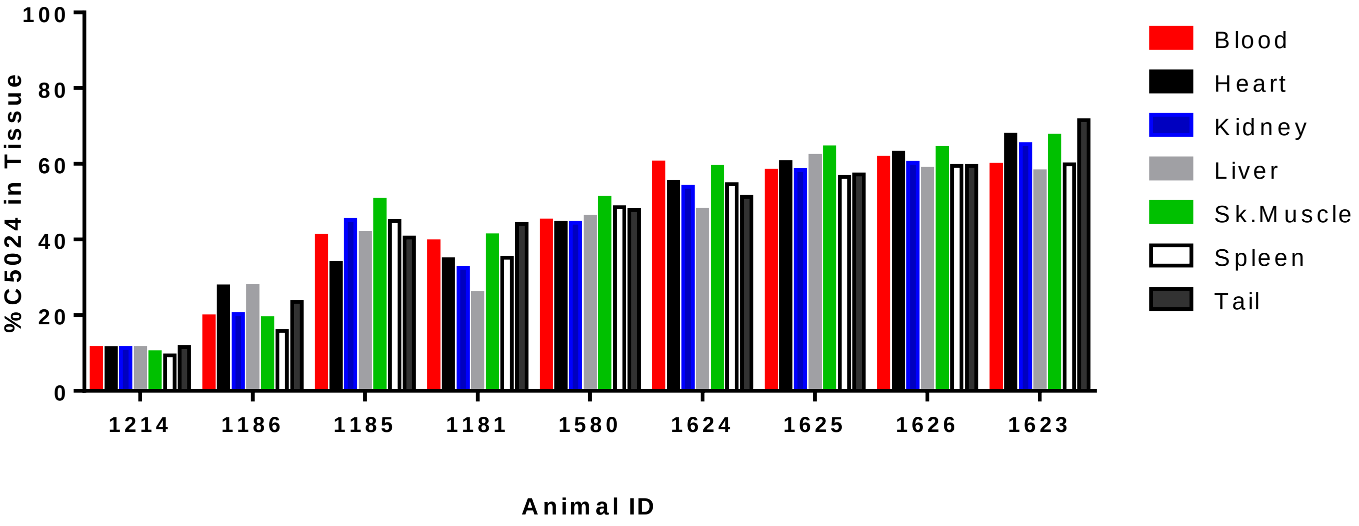

B

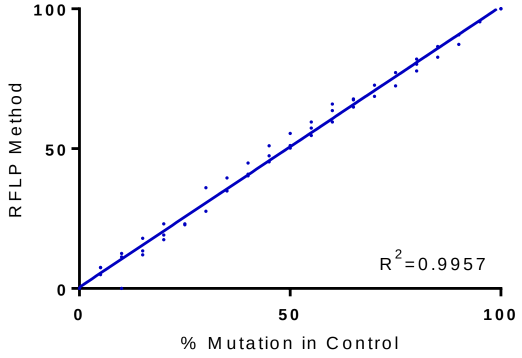

C

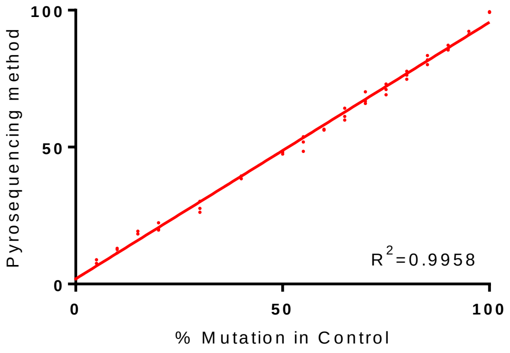

Supplemental Figure S5. Related to Figure 5.

(A) Limited tissue variation in mice with *tRNA*^{ALA} C5024T mutation in early life. Relative levels of the *tRNA*^{ALA} C5024T mutation measured in seven tissues from nine mice, at 20 weeks of age. At 20 weeks of age, there is little variation between levels in the blood and the levels from other tissues.

(B) Calibration curve for the RFLP assay to quantify the relative frequency of the *tRNA*^{ALA} C5024T.

(C) Calibration curve for the Pyrosequencing assay to quantify the relative frequency of the *tRNA*^{ALA} C5024T.

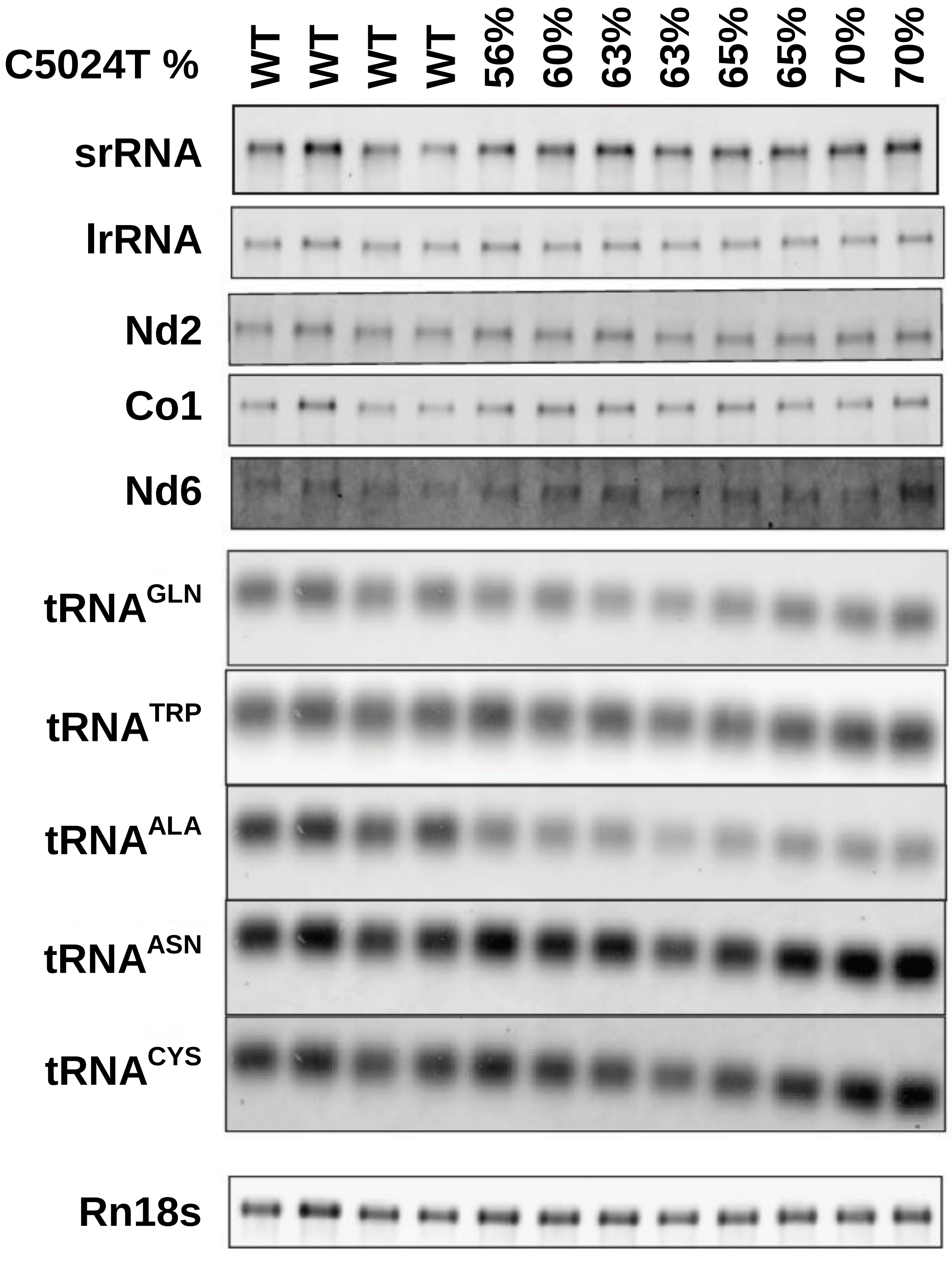

Supplemental Figure S6. Related to Figure 6.

tRNA^{ALA} steady-state level is strongly decreased in mice carrying high relative levels of the C5024T mutation. Representative northern blot of heart tissue with wild-type mice (WT) and mice that carry the *tRNA*^{ALA} C5024T mutation.

Supplemental Experimental Procedures

RFLP-PCR analysis of C5024T Mutation Load

An RFLP-PCR analysis method, similar to (Freyer et al., 2012) was developed. The C5024T mutation disrupts an HpyCH4III restriction site, which is used to differentiate the wild-type (270bp) fragment from the longer mutant fragment (360bp). The target mtDNA region was PCR amplified for 35 cycles at 58 °C annealing, using the primers CACTCATAGCAATAATAGCTC and CAGGAAACAGCTATGACCACAGTTTCGTAGGTTTAATTCCTGCC). The longer primer contains a control restriction site, which allows testing for incomplete digestion by the presence of a 380bp band in the assay.

After confirming amplification on an agarose gel, 2µl of additional PCR master mix including only the 6HEX labeled version of the short primer was added to the PCR mixture, and a single additional PCR cycle was carried out. The PCR mixture was purified using Agencourt AMPure XP - PCR Purification in a magnetic PCR tube holder. The DNA was eluted from the beads in 15 µl restriction enzyme buffer (1.5 µl Buffer 4, 0.5 µl HpyCH4III restriction Enzyme (NEB)) for >3 hours at 37°C. 2 µl of the digestion product was mixed with 0.1 µl of ROX 500 sizing ladder (ABI) and 7.9 µl of HiDi formamide, heated to 96 °C for 5 min and cooled directly on ice before separation on an ABI 3730 DNA analyzer using Fragment Analysis protocols (for 50 cm capillary array with POP7 polymer). Mother-offspring data from early generations was obtained by this method.

A calibration curve for the RFLP-PCR method was produced using using mixtures of plasmids containing clones of wild type and mutant sequence (Figure S5B).

Allelic Quantification Assay with Pyrosequencing

The second method utilized pyrosequencing technology on a PyroMark Q24 pyrosequencer (Qiagen). This Allele Quantification assay was developed using PyroMark assay design software v2.0 (Qiagen). A single PCR reaction was employed to amplify a 178 bp PCR fragment spanning the m.5024 mutation site, using a biotinylated forward primer and a non-biotinylated reverse primer (forward primer: 5'-Biotin-TTCCACCCTAGCTATCATAAGC, reverse primer: GTAGGTTTAATTCCTGCCAATCT). PCR products were combined with dH_2O, PyroMark binding buffer (Qiagen) and 1 µl Streptavidin sepharose TM high performance

beads (GE Healthcare), and purified and denatured using a Pyromark Q24 vacuum workstation (Qiagen). Sequencing was carried out with PyroMark Gold Q24 Reagents according to manufacturer's directions, using the sequencing primer (TGTAGGATGAAGTCTTACA). All dissected tissue sample data, microdissection quantification and later-generation mom-pup data was obtained using this method.

A calibration curves for this method was generated with a mixture of synthetic biotin-labelled oligos with wild type and mutant sequences (Figure S5B).
